# The Role of Glass Fiber-reinforced Composites in Maxillary Fracture Repair

**DOI:** 10.31661/gmj.v13i.3520

**Published:** 2024-09-25

**Authors:** Ashkan Badkoobeh, Elahe Mozafari Ghadikolaei, Seyed Mohammad Mahdi Mirmohammadi, Seyad Ayub Tabatabaie Mayanie, Zahra Bahman, Naghmeh Shenasa, Sajad Raeisi Estabragh

**Affiliations:** ^1^ Department of Oral and Maxillofacial Surgery, School of Dentistry, Qom University of Medical Sciences, Qom, Iran; ^2^ Faculty of Dentistry, Baku Medical University, Baku, Azerbaijan; ^3^ Department of Oral and Maxillofacial Surgery, Beheshti Medical University, Tehran, Iran; ^4^ Faculty of Dentistry, Belarusian State Medical University, Minsk, Belarus; ^5^ Department of Endodontics, Shahrekord University of Medical Sciences, Shahrekord, Iran; ^6^ Department of Prosthodontics and Oral and Dental Diseases Research Center, Kerman University of Medical Sciences, Kerman, Iran

**Keywords:** Maxillary Fracture, Glass Fiber Reinforcement, Repair Techniques, Maxillofacial Surgery

## Abstract

Maxillary fractures present complex challenges in facial trauma repair due to the intricate anatomy and functional importance of the midface. Traditional fixation methods, such as titanium plates and screws, provide mechanical stability but are associated with complications, including infection, palpability, and interference with imaging. This review examines the role of Glass Fiber-reinforced Composites (GFRC) as an emerging alternative for maxillary fracture repair, emphasizing its mechanical properties, clinical applications, and potential for improving patient outcomes.GFRC offers distinct advantages, including high tensile strength, flexibility, and biocompatibility. These properties enable more effective stress distribution across the fracture site, reducing localized pressure and enhancing bone healing. GFRC’s radiolucency and lightweight nature also address aesthetic concerns, as it eliminates the visibility and palpability issues commonly associated with metallic implants. This review compares GFRC to traditional materials such as titanium and composite resorbable polymers, highlighting its superior performance in terms of mechanical stability, patient comfort, and long-term durability. The review also explores emerging technologies in GFRC, such as bioactive coatings and nanotechnology, which have the potential to enhance its biological integration and promote faster bone regeneration.

## Introduction

Maxillary fractures, often resulting from trauma or injury to the facial skeleton, pose significant clinical challenges due to the intricate anatomy and functional importance of the midface [1][2][3]. These fractures, if not treated adequately, can lead to severe complications, including impaired mastication, speech, and aesthetics, ultimately affecting the patient’s quality of life [1][4]. Traditional approaches to maxillary fracture repair, such as the use of metal plates, screws, and wire fixations, have been widely employed with varying success rates [5]. While these methods provide mechanical stability, they often come with drawbacks such as bulkiness, risk of infection, and the need for secondary surgeries to remove hardware [6][7].

Due to these challenges, the search for more biocompatible, lightweight, and durable materials has led to the exploration of advanced biomaterials in maxillofacial surgery [8]. Over the years, several reinforcement materials have been developed, including autologous bone grafts, allografts, and various synthetic polymers [6][9]. However, in recent years, fiber-reinforced materials have gained significant attention due to their potential to improve mechanical strength without compromising the biological compatibility of the repair [10].

Glass Fiber-reinforced Composites (GFRC), originally used in dental applications such as orthodontics and prosthodontics, has emerged as a promising material in the field of maxillofacial surgery [11][12]avoidance of periodontal disease and interproximal caries. A baseline examination was performed and the patients were examined regularly at six-month intervals (nine years’ follow-up. Its combination of lightweight properties, biocompatibility, and high tensile strength make it an attractive alternative to traditional metallic and polymer-based fixations [13][14]. Moreover, GFRC’s ability to integrate with surrounding tissues without provoking adverse reactions makes it particularly suited for complex craniofacial structures like the maxilla [15].

This review aims to provide a comprehensive examination of the role of GFRC in the repair of maxillary fractures. The scope of this article includes an overview of traditional and modern fracture repair techniques, an in-depth analysis of the biomechanical properties of GFRC, and its clinical applications in maxillary fractures.

## Maxillary Fractures: Clinical Presentation and Challenges

Maxillary fractures are a common result of trauma to the midface, often caused by incidents such as road traffic accidents, sports injuries, falls, and physical assaults [16][17]. The maxilla, being a central structure in the facial skeleton, plays a crucial role in supporting the orbit, nasal cavity, and upper dental arch [18][19]. Therefore, fractures of the maxilla not only affect facial aesthetics but also impair essential functions such as speech, mastication, and breathing [4][19]. Depending on the location and severity of the injury, maxillary fractures can range from isolated infraorbital fractures to more complex Le Fort fractures, which involve the entire midface. These fractures are typically classified into three types, known as Le Fort I, II, and III, based on the pattern of fracture lines and the associated displacement of bone [20][21].

The clinical presentation of maxillary fractures varies depending on the type and extent of the injury. Common symptoms include facial swelling, malocclusion (misalignment of teeth), numbness due to nerve damage, and, in severe cases, mobility of the upper jaw [17][22]. In addition, these fractures can disrupt the normal alignment of the orbit, leading to visual disturbances, while nasal involvement often causes breathing difficulties [23]. Given the complex anatomy and the functional importance of the maxilla, the proper management of these fractures is critical for restoring both aesthetics and functionality [19].

One of the primary challenges in treating maxillary fractures is achieving adequate mechanical stability while maintaining the integrity of the surrounding soft tissues and facial structures [2][8]. Traditional fixation methods, such as titanium plates and screws, are effective in providing stability but can sometimes be associated with complications [2]. Metal plates, while strong, may lead to palpability beneath the skin, interfere with imaging studies, and, in some cases, cause thermal sensitivity or cold intolerance [24]. Additionally, hardware infection is a persistent risk, particularly in cases involving poor wound healing or secondary trauma. The possibility of long-term foreign body reactions and the need for a second surgery to remove hardware add to the complexity of care [2][25].

Infection risk is another critical concern in maxillary fracture repair, especially in cases where the fracture communicates with the nasal or oral cavity [26]. The high bacterial load in these areas can increase the likelihood of postoperative infections, necessitating careful management with antibiotics and thorough debridement during surgery [27]. Furthermore, maxillary fractures can disrupt the blood supply to the bone, complicating the healing process and increasing the risk of non-union or delayed healing [28].

Aesthetic considerations also play a significant role in the management of maxillary fractures [29]. The maxilla forms the foundation of the midface, and any malalignment or improper fixation can lead to visible deformities, such as facial asymmetry or sunken cheeks [29][30]. Restoring facial contours while ensuring proper dental occlusion is often a delicate balance, requiring meticulous surgical planning and execution [30].

Given these challenges, the use of reinforcement materials that offer both mechanical strength and biocompatibility has become increasingly important in maxillary fracture repair.[10][24] GFRC, in particular, presents a promising alternative, providing sufficient rigidity for stabilization while being lightweight and biocompatible [31]. Unlike metal plates, glass fiber is non-reactive and can be integrated more easily with surrounding tissues, potentially reducing the risk of infection and foreign body complications [15][32]. Additionally, its translucent appearance helps to minimize aesthetic concerns, making it an attractive option for treating complex maxillary fractures [33].

## GFRC: Properties and Advantages

GFRC, a material long utilized in various engineering fields, has recently found significant applications in medical and dental fields, particularly in the reinforcement of bone structures [15][34]. GFRCs are composed of fine, hair-like strands of glass that are woven or bundled together to create materials with exceptional mechanical properties [35]. The material's combination of strength, flexibility, and biocompatibility makes it a promising alternative to traditional metallic and polymer-based reinforcements in maxillofacial surgery [12][32].

One of the primary characteristics of GFRC is its high tensile strength, which refers to its ability to resist forces that attempt to pull it apart [35]. The tensile strength of GFRC can range between 1,200 and 3,400 MPa, depending on the specific type and manufacturing process [36]. This property ensures that GFRC can endure significant stress before failing, making it suitable for reinforcing fractures in load-bearing structures like the maxilla [37][38]. In comparison to metals such as titanium, GFRC is considerably lighter, reducing the overall weight of the fixation materials and minimizing the burden on the healing bone [24].

GFRC also offers considerable flexibility. Unlike rigid metallic plates, GFRCs can be woven into various configurations, allowing them to conform to the complex, curved anatomy of the maxilla [24][39]. This flexibility provides surgeons with greater versatility in shaping the reinforcement to fit specific fracture patterns, contributing to a more natural reconstruction of the facial structure [33]. Moreover, the inherent flexibility of GFRC helps distribute stress more evenly across the fracture site, reducing the risk of localized pressure points that could lead to complications such as bone resorption or implant failure [15][34].

In terms of biocompatibility, GFRC has a well-established track record in medical applications [31]. The material is chemically inert, meaning it does not interact with surrounding tissues or degrade over time, which significantly lowers the risk of adverse reactions, such as inflammation or foreign body responses [15][40]. This biocompatibility is a crucial advantage in maxillofacial applications, where the proximity of delicate structures such as the sinus cavities, nerves, and mucosal tissues requires materials that do not provoke irritation or excessive scarring [31].

## GFRCs vs. Other Materials

**Table:** Table1. Comparison of GFRC with Other Reinforcement Materials

		**Materials **		
**Property**	**GFRC**	**Carbon Fiber Reinforcement **	**Titanium Reinforcement **	**Biodegradable Polymers **
**Tensile Strength**	High tensile strength, effective for load-bearing applications.	Very high tensile strength, often higher than GFRCs.	Extremely high tensile strength, considered the gold standard for strength.	Lower tensile strength, not suitable for high load-bearing applications.
**Flexural Strength**	High flexural strength, suitable for complex anatomical structures.	Excellent flexural strength, but more brittle than GFRCs.	Excellent flexural strength, highly durable.	Lower flexural strength, generally used in non-load-bearing areas.
**Biocompatibility**	Excellent biocompatibility with minimal adverse reactions.	Good biocompatibility, but potential for long-term degradation issues.	Good biocompatibility, but risk of metal allergies in some patients.	Excellent biocompatibility, with the added benefit of resorption.
**Corrosion Resistance**	Superior resistance to corrosion in biological environments.	Good corrosion resistance, but not as high as GFRCs.	Excellent corrosion resistance, particularly in saline environments.	Good corrosion resistance, as they are designed to degrade over time.
**Cost**	Moderate to high cost.	High cost, often more expensive than GFRCs.	Moderate to high cost, depending on the specific alloy.	Moderate cost, generally cheaper than GFRCs and metals.
**Imaging Compatibility**	Compatible with imaging modalities such as MRI and CT.	Generally compatible with imaging, but can cause artifacts in some cases.	Can cause significant artifacts in MRI and CT scans.	No interference with imaging modalities.
**Aesthetic Outcome**	Improved aesthetic outcomes due to reduced visibility under the skin.	Moderate aesthetic outcomes, depending on visibility.	Poor aesthetic outcomes due to visibility and palpability under the skin.	Excellent aesthetic outcomes as they are resorbed and leave no trace.
**Reference**	[24]	[42]	[41]	[43]

Table-1 compared the GFRC with other reinforcement materials. GFRC offers several advantages over traditional materials like titanium in maxillary fracture repair, making it a superior choice for both functional and aesthetic outcomes [24]. While titanium is strong, it often causes issues such as cold sensitivity, infection risks, and interference with imaging techniques like CT scan. In contrast, GFRC's radiolucency allows for clearer postoperative imaging without metallic artifacts, making it easier for clinicians to monitor healing [41]. It is also less likely to be visible or palpable under the skin, which is important in facial surgeries where aesthetics play a key role [30]. Additionally, GFRC’s resistance to corrosion and chemical degradation ensures durability and longevity in the body, reducing the need for secondary surgeries to remove implants [44]. Its lightweight and flexible nature makes it more comfortable for patients, especially in complex fractures where reinforcement needs to adapt to intricate bone structures [45].

Although carbon fiber reinforcement shares similarities with GFRC, it falls short in several critical areas. Carbon fiber is less biocompatible, which increases the risk of adverse tissue reactions when used in medical applications [42]. Additionally, its resistance to corrosion is inferior compared to GFRC, making it less durable for long-term use in the body [46]. From an aesthetic standpoint, carbon fiber is more visible under the skin and does not offer the same translucency as GFRC, which can be a disadvantage in facial surgeries where appearance is important [42]. Furthermore, carbon fiber tends to be more expensive, making it a less cost-effective option for maxillofacial applications. This combination of lower biocompatibility, reduced corrosion resistance, and poorer aesthetic outcomes, along with its higher cost, makes carbon fiber a less favorable choice compared to GFRC for maxillary fracture repairs and similar medical procedures [45].

## Clinical Applications and Techniques

The use of GFRC in maxillary fracture repair has advanced significantly, offering clinicians an alternative to traditional metallic fixation methods. The application of GFRC in clinical settings primarily revolves around two primary techniques: pre-impregnated GFRC sheets and customized splints, each tailored to address the unique challenges of maxillary fractures [47][48].

Techniques for Using GFRC in Maxillary Fracture Repair

One common technique involves the use of pre-impregnated GFRC sheets, which are prefabricated materials that come impregnated with a resin matrix. These sheets can be easily molded to conform to the intricate geometry of the maxilla, allowing for precise adaptation to various fracture patterns [47]. The flexibility of these sheets enables surgeons to apply them over curved or irregular surfaces without the need for extensive manipulation [49]. Once positioned, the resin is light-cured or chemically activated, hardening the GFRC into a rigid structure that provides immediate stability to the fracture site [47].

Another approach is the use of customized GFRC splints, which are fabricated based on a patient’s specific fracture morphology [48]. These splints are particularly useful for Le Fort fractures, where multiple planes of the maxilla are involved. Customized splints are designed using 3D imaging and computer-assisted design (CAD) technology, ensuring an exact fit for the patient [50][51]. During surgery, these splints are fixed in place using biocompatible adhesives or additional fixation devices, allowing for both functional support and aesthetic reconstruction [48]. The translucency of GFRC ensures that the splint remains virtually invisible under the skin, addressing one of the major aesthetic concerns associated with traditional metallic fixations [31][52].

In both techniques, the light weight of GFRC and its ability to distribute loads evenly across the fracture site provide an ideal environment for bone healing, reducing the risk of complications such as implant failure or malocclusion [47][48]. These techniques also avoid the drawbacks of metallic plates, such as palpability and interference with imaging modalities like CT scans or MRIs, as GFRC is radiolucent [53].

Case Studies and Clinical Trials

Several case studies and clinical trials have demonstrated the effectiveness of GFRC in maxillary fracture repair, showcasing its versatility and patient outcomes.

GFRCs have been shown to improve fracture resistance, with studies like that of Pang et al. [51] demonstrating enhanced fracture resistance in restorations using CAD/CAM technology. Furthermore, Khidr et al [48]. illustrated the advantages of using fiber-reinforced splints in pediatric maxillofacial fractures, showing high aesthetic satisfaction and minimal complications. Both studies highlight the success of GFRC splints in reducing infection risks and providing better patient comfort compared to traditional metal systems [48][51]. These findings reinforce the benefits of GFRCs for surgical outcomes in complex facial fractures, particularly with improved aesthetics and fewer implant-related complications.

Moreover, Longeac et al. [54] demonstrated the benefits of utilizing virtual surgical planning and 3D models for the precise treatment of zygomaticomaxillary complex fractures. This method allows for enhanced accuracy in fracture reduction and improves both mechanical stability and aesthetic outcomes. Similarly, research by Schneider et al. [55] emphasizes the importance of incorporating advanced materials in zygomaticomaxillary complex repairs, highlighting their role in improving facial symmetry and minimizing postoperative complications [54][55]. These innovations in surgical planning and material science contribute to better long-term patient outcomes.

Several studies show that GFRCs provide enhanced fracture resistance and superior outcomes in restorative dentistry applications, offering advantages such as reduced postoperative complications and lower risks of infections compared to traditional materials like titanium [24][56][57].

Also, Ranjkesh et al. [44] demonstrated significant patient-friendly benefits, including reduced hardware removal procedures due to the biocompatibility and lightweight nature of GFRCs. These findings suggest that GFRCs may offer a more patient-friendlier and effective alternative to titanium plates in certain clinical contexts.

Complications and Limitations

Despite the promising results, there are certain complications and limitations associated with the use of GFRC in maxillary fracture repair [58]. One of the primary concerns is the risk of fracture propagation. While GFRCs are known for their ability to resist tension and prevent crack propagation, studies indicate that over time, fractures in the composite mass may still develop, particularly under high occlusal loads or in the presence of repeated stress [59]. This presents a significant challenge for long-term stability in maxillary fracture repair, where the material is subjected to constant pressure and movement in the oral environment.

Another major limitation of GFRC is its sensitivity during handling and placement. The success of a GFRC restoration relies heavily on precise application techniques, and errors during the bonding process can lead to weakened structures. Inadequate bonding may increase the risk of failure, particularly in areas subjected to high mechanical stress, such as the maxillary region [60]. Additionally, complications such as chipping or fracturing of the composite layers are common, especially when high occlusal forces are involved. This not only reduces the longevity of the repair but can also necessitate further intervention, increasing the risk of subsequent complications [61].

Moreover, although GFRC performs well in laboratory settings, it’s in vivo clinical performance may not always align with these results [62]. The mechanical properties of GFRC are not universally applicable for all types of maxillary fractures, especially in cases of complex or severe fractures [63]. Studies have also raised concerns about the challenges in repairing GFRC restorations once they fail, as repaired materials often do not regain the same strength and durability as the original composite [64][65][66].

Lastly, cost may also be a factor limiting widespread adoption [63]. Although GFRCs offer numerous benefits over traditional metal plates, they are often more expensive, particularly when customized splints are fabricated using advanced CAD technology [67]. This could make them less accessible in certain healthcare settings, especially in regions where healthcare resources are limited [68].

## Emerging Technologies in GFRC

Several emerging technologies are shaping the future of GFRC, particularly in terms of material enhancements and improved integration with biological tissues.

Nanotechnology in GFRCs:

1. Nanotechnology has the potential to significantly advance the properties of GFRCs [69]. By incorporating nanoparticles or nanofibers into GFRCs, researchers can enhance the mechanical properties, such as tensile strength, toughness, and fatigue resistance [70]. Nanomaterials can improve the bonding between GFRCs and the resin matrix, creating stronger and more resilient composites [71].

2. Nanofibers are also being explored for their ability to mimic the extracellular matrix, which could improve cellular adhesion and promote bone regeneration at the fracture site [72]. These nanofibers can be integrated into GFRCs to provide a scaffold that supports the natural healing process, making the material more bioactive and conducive to bone growth [73].

Bioactive GFRC:

1. Bioactive glass, a material that promotes the formation of hydroxyapatite (a mineral that is a key component of bone), could be incorporated into GFRCs [72]. Bioactive coatings or modifications to the GFRC itself can stimulate the bone-forming process, accelerating healing and improving the integration of the implant with the surrounding bone tissue [74][75].

2. Silicate-based bioactive glasses have already been shown to induce osteogenesis and angiogenesis, processes critical to bone healing [76]. When applied to GFRCs, these materials could actively promote bone regeneration, making the fracture repair process faster and more reliable [74].

3. Future bioactive GFRCs may also include growth factor delivery systems that release molecules such as bone morphogenetic proteins (BMPs) [77] or vascular endothelial growth factor (VEGF), which could further accelerate healing by stimulating bone and blood vessel formation [78].

Hybrid Glass Fiber-polymer Systems:

1. Hybrid systems that combine GFRC with resorbable or non-resorbable polymers are being actively explored as a way to balance mechanical stability with tissue integration [70]. These composite systems can provide initial strength while gradually transferring load to the healing bone as the polymer degrades [71].

2. Innovations in 3D printing technology could also enable the customization of glass fiber-polymer composites, allowing for patient-specific designs that are tailored to the anatomy and fracture pattern of each individual [79]. 3D-printed GFRC implants could offer unprecedented precision and fit, improving the overall success of maxillary fracture repairs [50].

## Conclusion

This comprehensive review has highlighted the significant role of GFRC in maxillary fracture repair, demonstrating its unique advantages over traditional materials such as metals, composites, and resorbable polymers. GFRC offers exceptional mechanical properties, including high tensile strength, flexibility, and load distribution, which are critical for maintaining stability in the complex anatomy of the maxilla. Its biocompatibility, radiolucency, and aesthetic superiority further distinguish it from conventional materials, reducing complications such as infection, palpability, and hardware removal surgeries.

Comparative studies have shown that GFRC promotes more effective fracture healing, enhances patient comfort, and provides long-term durability, making it a valuable tool for clinicians. The material’s ability to evenly distribute stress and integrate well with bone tissue supports faster and more reliable recovery. Moreover, emerging technologies such as nanotechnology, bioactive coatings, and hybrid systems offer exciting prospects for future innovations, potentially transforming GFRCs into a more interactive and dynamic tool in maxillofacial surgery.

## Conflict of Interest

The authors declare no conflict of interest.
